# A modified closure method during endoscopic full-thickness resection for a gastrointestinal stromal tumor: “neck” clipping and cutting

**DOI:** 10.1055/a-2178-4629

**Published:** 2023-11-20

**Authors:** Zeliang Yang, Yuting Jiang, Yangyang Chen, Jie Yang, Xiaoling Zheng

**Affiliations:** 1 74551 Shengli Clinical Medical College, Fujian Medical University, Fuzhou, China; 2 117861 Digestive Endoscopy Department, Fujian Provincial Hospital, Fuzhou, China

Endoscopic full-thickness resection (EFTR) is a surgical approach to effectively resect gastrointestinal stromal tumors (GISTs) that originate from the intrinsic muscular layer and grow in a convex fashion toward the peritoneal cavity. Because of the intentional perforation formed by EFTR, the patient's prognosis is closely related to preventing gastrointestinal contents entering the extraluminal space. In pursuit of the most effective closure methods, different endoscopic closure techniques are therefore being developed. An endoscopic closure method was first developed by Zhou et al. using metal clips to close the post-EFTR defect, which was later developed with the endoloop-assisted closure method, and finally emerged as various methods including the over-the-scope (OTS) clip method 1 , all with the ultimate goal of pursuing the fastest closure to prevent the passage of gastrointestinal contents and ultimately improve the patientʼs prognosis.

During EFTR procedures, it is particularly important to avoid tumors falling into the abdominal cavity, especially mesenchymal tumors, where the main body is convex to the outside of the cavity. We combined an endoscopic suspension traction technique with a modified endoscopic closure to achieve tumor removal and rapid closure during an endoscopic procedure ( Video 1 ).

A modified closure method for the defect after endoscopic full-thickness resection for a gastrointestinal stromal tumor that achieves tumor removal and rapid closure.Video 1

In this case, for the first time, a full-thickness resection was performed around the tumor and one-tenth of the mucosa on the oral side was reserved to form a “neck” between the tumor and the defect by traction suspension. During traction, the mucosa near the “neck” formed a narrow acute angle, which made applying clips to the mucosa on both sides easy, so the clips could be quickly positioned and placed onto the most effective tissue for closure. ( Fig. 1 )

**Fig. 1:**
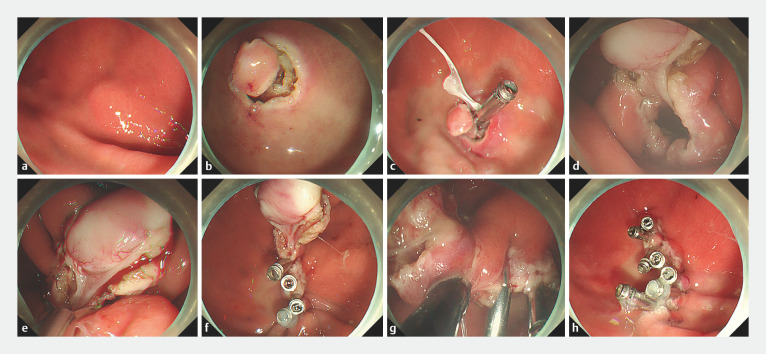
Endoscopic views during an endoscopic full-thickness resection procedure showing: **a** the lesion; **b** a pre-incision made around the tumor after marking; **c** suspension of the tumor with the aid of traction; **d** full-thickness resection around the tumor with one-tenth of the mucosa on the oral side left to create a “neck”; **e,f** initial closure performed by lifting the tumor and closing the defect closest to the “neck”; **g** excision of the mucosa of the “neck”; **h** closure of the remaining mucosa by metal clips.

First, the lesion was marked and a pre-incision was made around the lesion to expose the tumor ( Fig. 1 **a,b** ). The tumor was then suspended with the aid of traction using dental floss and a metal clip ( Fig. 1 **c** ). Third, full-thickness resection around the tumor was performed, leaving one-tenth of the mucosa on the oral side ( Fig. 1 **d** ). Next, the first part of the closure was started by lifting the tumor and closing the defect closest to the “neck,” before proceeding with defect closure from the oral to the anal side ( Fig. 1 **e,f** ). The mucosa of the “neck” was then excised to complete the dissection of the tumor ( Fig. 1 **g** ). The remaining mucosa was closed by metal clips to complete the final closure ( Fig. 1 **h** ).

In conclusion, this improvement of the closure method is designed to effectively improve the speed of defect closure during EFTR, thereby hopefully improving the patient prognosis; however, further prospective studies are needed to investigate patient prognosis.

Endoscopy_UCTN_Code_TTT_1AO_2AG

## Notes

Endoscopy E-Videos https://eref.thieme.de/e-videos
*E-Videos* is an open access online section of the journal *Endoscopy* reporting on interesting cases and new techniques in gastroenterological endoscopy. All papers include a high-quality video and are published with a Creative Commons CC-BY license. Endoscopy E-Videos qualify for HINARI discounts and waivers and eligibility is automatically checked during the submission process. We grant 100% waivers to articles whose corresponding authors are based in Group A countries and 50% waivers to those who are based in Group B countries as classified by Research4Life (see: https://www.research4life.org/access/eligibility/ ). This section has its own submission website at https://mc.manuscriptcentral.com/e-videos .
